# Gut microbial signatures in schizophrenia: exploring archaea, fungi, and bacteria

**DOI:** 10.1186/s12888-025-07721-3

**Published:** 2026-01-02

**Authors:** Rui Fu, Xue-jun Liang, Wen-mao Yang, Rui Li, Yan-ru Shi, Li Guo, Huan Yu, Yi-huan Chen, Hua-ning Wang

**Affiliations:** 1 Department of Psychiatry, Xijing Hospital, Fourth Military Medical University, Xi’an, 710032 China; 2 Department of Psychiatry, Chang’an Hospital, Xi’an, 710000 China; 3 Mental Diseases Prevention and Treatment Institute of Chinese PLA, No.988 Hospital, Jiaozuo, 454003 China

**Keywords:** Schizophrenia, Gut microbiota, Archaea, Fungi, Bacteria

## Abstract

**Background:**

Gut microbial, mainly bacterial dysbiosis, has been demonstrated in patients with schizophrenia (SCH). However, the signatures and differences of minority gut microbiota in SCH, such as archaea and fungi, have been poorly addressed.

**Methods:**

We obtained stool samples from 61 SCH patients and 69 healthy controls (HC), and analyzed the compositional and functional alterations of gut archaea, fungi, and bacteria using metagenomic shotgun sequencing (MSS). Additionally, we developed potential biomarkers to distinguish SCH from HC.

**Results:**

SCH patients showed significantly lower archaeal α-diversity compared with that of HC. Whereas there were significant differences between SCH and HC in β-diversity at the species level of archaea, fungi and bacteria. Meanwhile, the functional differences between the two groups were concentrated in glucose, lipid and amino acid metabolic pathways. Furthermore, we established potential diagnostic archaeal (9 species, AUC = 0.73), fungal (8 species, AUC = 0.69), and bacterial (22 species, AUC = 0.74) microbiomes for differentiating SCH patients from HC.

**Conclusions:**

This study describes a more comprehensive understanding of abnormal gut microbiome in SCH and might provide candidate targets for the development of a microbe-based diagnosis for SCH.

**Trial registration:**

Chinese Clinical Trial Registry: ChiCTR2000032118, registration date: 2020/04/20.

**Supplementary Information:**

The online version contains supplementary material available at 10.1186/s12888-025-07721-3.

## Introduction

Schizophrenia (SCH) is a highly complex and heritable psychiatric condition, marked by behavioral abnormalities and cognitive impairments [1]. The commonly cited prevalence of SCH indicates that approximately 1% of the population will develop the disorder over their lifetime, with an equal distribution between males and females [2]. SCH is associated with considerable physical and social morbidity, and its diagnostic criteria are primarily based on a constellation of positive, negative, and disorganized symptoms, where disorganized symptoms refer to disordered thinking, speech, and behavior (e.g., loose associations, tangentiality, incoherent conversation) [3]. However, these core symptoms are often accompanied by various additional features, such as movement disorders, affective disturbances, and sensory abnormalities [4, 5]. Among individuals who meet the diagnostic criteria, there is significant variability in symptom presentation, onset, course, and outcome [6, 7]. Moreover, the symptomatology and genetics of SCH frequently intersect with those of other psychiatric conditions, making precise boundaries difficult to delineate [8, 9]. For example, there is substantial symptom overlap with bipolar disorder and major depressive disorder [10, 11]. Additionally, antipsychotic agents, despite being the mainstay of current pharmacological treatment, are often ineffective in addressing negative and disorganized symptoms. They fail to manage psychosis in approximately 30% of cases adequately and are associated with numerous adverse effects [12, 13]. Hence, there is a pressing need for therapeutic advancements. However, progress has been hindered by an incomplete understanding of the underlying pathophysiology, clinical heterogeneity, and the absence of reliable biomarkers for SCH [14].

The gut microbiota serves as a crucial mediator in the bidirectional communication between the digestive system and the central nervous system (CNS), thus constituting the microbiota-gut-brain axis (MGBA) [15]. It affects neural function directly through the vagus nerve and indirectly via the endocrine function, neurotransmitter synthesis, immune system and other signaling molecules [16, 17]. Alterations in the gut microbiota and its products have been implicated in neurological and psychiatric disorders [18, 19], and recent independent research has extended these associations to SCH [20]. For example, the microbial composition in individuals with SCH, including those who are untreated, differs significantly from that of matched healthy controls [21, 22]. Furthermore, fecal microbiota transfer (FMT) from SCH patients induces SCH-related behaviors in germ-free (GF) recipient mice [23]. Similarly, the transplantation of *Streptococcus vestibularis* ATCC 49,124, a bacterium enriched in SCH patients, induces social behavior deficits and neurotransmitter alterations in recipient mice [24]. These studies elucidate the pathogenesis of schizophrenia from a perspective beyond neurotransmitter abnormalities and lay the groundwork for therapeutic strategies based on microbial interventions [25]. Nevertheless, up to now, the majority of studies have predominantly focused on the bacterial composition of the gut microbiota in SCH, revealing a range of alterations in microbial diversity and composition among affected individuals [26, 27]. There remains a significant knowledge gap concerning the composition and function of minority gut microbiota in SCH, such as fungi and archaea, which also play a role in intestinal balance and function. Future research aims to address this gap and might provide a more comprehensive understanding of the gut microbiota’s role in SCH pathogenesis.

The metagenomic shotgun sequencing (MSS) coupled with bioinformatics tools not only facilitates the investigation of a broader spectrum of microbial communities, encompassing bacteria, archaea, and fungi, but also enhances the detailed characterization of these microbiota [28, 29]. In this study, we conducted MSS analyses of gut archaea, fungi, and bacteria on 130 samples, comprising 61 cases and 69 healthy controls (HC). The functional alterations in the gut microbiota associated with SCH were elucidated through pathway/module analysis based on the Kyoto Encyclopedia of Genes and Genomes (KEGG). Additionally, we developed potential biomarkers derived from archaea, fungi, bacteria, and their combinations to distinguish SCH from HC.

## Methods

### Participant

Participants with SCH and HC individuals were recruited from the Department of Psychiatry at Chang’an Hospital between June 2020 and August 2023. The study was approved by the Ethics Committee of the Chinese Clinical Trial Registry (ChiECRCT20200090), and registered with the Chinese Clinical Trial Registry (ChiCTR2000032118). All potential participants received comprehensive information regarding the study’s purpose, procedures, risks, and benefits. Written informed consent was obtained from each participant, confirming their voluntary participation. Diagnostic and screening criteria: Participants in the SCH group were required to meet the diagnostic criteria for schizophrenia as outlined in the Diagnostic and Statistical Manual of Mental Disorders, Fifth Edition (DSM-5). The Mini-International Neuropsychiatric Interview (MINI) was employed to identify any pre-existing psychiatric disorders [30]. The Positive and Negative Syndrome Scale (PANSS) was administered to assess symptom severity, with a total score of ≥ 60 required for inclusion [31, 32]. Participants in the HC group were screened using a semi-structured clinical interview to exclude individuals with any current or past psychiatric disorders. Medical history and physical examination results were reviewed to exclude individuals with any significant physical illnesses. All participants were required to be between the ages of 18 and 65 years. Participants were excluded if they had taken probiotics, antibiotics, prebiotics, or probiotic-fermented foods (such as yogurt) within one month prior to enrollment. This criterion was essential to minimize potential confounding effects on the study outcomes. The following exclusion criteria were applied to both groups to ensure the homogeneity of the study population and to reduce possible biases: Presence of gastrointestinal diseases (e.g., Crohn’s disease, pancreatitis); obesity (body mass index (BMI) ≥ 28.0); hyperlipidemia (triglycerides ≥ 2.3 mmol/L); hypertension (systolic BP ≥ 140 mmHg or diastolic BP ≥ 90 mmHg); hyperglycemia (FBG ≥ 6.1 mmol/L or 2 h TC ≥ 7.8 mmol/L). Individuals diagnosed with diabetes or undergoing diabetes treatment; Severe dietary imbalances, such as a preference for high-fat diets or long-term vegetarianism; Pregnant or breastfeeding women; Presence of other psychiatric disorders according to the DSM-5 diagnostic criteria; Individuals who had taken any type of psychotropic medication in full doses for more than two consecutive days within two weeks before the start of the study. On the day of fecal sample collection, all participants underwent assessments. Detailed medical histories, physical examination findings, and laboratory test results were documented for each individual.

### DNA extraction

Genomic DNA was extracted from 0.5 g of stool using the PF Mag-Bind Stool DNA Kit (Omega Bio-tek, Norcross, GA, U.S.) following the manufacturer’s protocol. DNA concentration was measured with a TBS-380 fluorometer, and purity was assessed using a NanoDrop 2000 spectrophotometer. DNA quality was verified via 1% agarose gel.

### Metagenomic sequencing

DNA was fragmented to an average size of approximately 400 bp using the Covaris M220 (Gene Company Limited, China) for library construction. The paired-end library was then constructed with the NEXTFLEX Rapid DNA-Seq Kit (Bioo Scientific, Austin, TX, USA). Paired-end sequencing was performed on Illumina Novaseq 6000 (Illumina Inc., San Diego, CA, USA) at Majorbio Bio-Pharm Technology Co., Ltd. (Shanghai, China) using NovaSeq 6000 S4 Reagent Kit according to the manufacturer’s instructions (www.illumina.com).

### Processing of metagenome sequencing data

The data were analyzed using the Majorbio Cloud Platform (www.majorbio.com). Briefly, raw sequencing reads were processed with fastp (version 0.20.0) to trim adaptors and remove low-quality reads (length < 50 bp, quality value < 20, or containing N bases) [33]. Reads were then aligned to the human genome using BWA (version 0.7.17), and any hits associated with the reads and their mates were removed [34]. The quality-filtered data were assembled with MEGAHIT (version 1.1.2), and contigs ≥ 300 bp were selected [35]. Open reading frames (ORFs) were predicted from the assembled contigs using Prodigal (version 2.6.3), and ORFs ≥ 100 bp were retrieved [36]. A non-redundant gene catalog was constructed with CD-HIT (version 4.7) at 90% sequence identity and 90% coverage [37]. Gene abundance for each sample was estimated using SOAPaligner (version soap2.21release) with 95% identity [38].

### Taxonomic and functional annotation

The best-hit taxonomy of non-redundant genes was determined by aligning them against the NCBI NR database using DIAMOND (version 2.0.11) with an e-value cutoff of 1e-5 [39]. Similarly, functional annotation (KEGG) was performed. Subsequently, differential analysis was conducted at taxonomic, functional, and gene levels based on the abundance profiles of non-redundant genes using Linear Discriminant Analysis Effect Size (LEfSe).

### Statistical analysis

Statistical analyses were conducted using R-3.5.3 and SPSS 21.0. Gender differences were compared using chi-squared analysis. Continuous variables were assessed for normality using the Shapiro–Wilk test. Normally distributed data were presented as mean ± SD, while non-normally distributed data were represented by median (M) and interquartile range (*P*_25_, *P*_75_) and analyzed using the Wilcoxon or Mann–Whitney U test. The multiple test correction was performed using the False Discovery Rate (FDR) method. Spearman’s rank correlation coefficient was calculated in the R heatmap package (version 3.3.1) to assess the relationship between clinical variables and species. A receiver operating characteristic (ROC) analysis was performed using SPSS 19.0, and ROC curves were generated using GraphPad Prism 9.0. The significance level was set at α = 0.05 for two-tailed *P* values.

## Results

### Clinical and sequencing characteristics of the recruited participants

Initially, 72 patients with SCH and 78 healthy controls (HC) were enrolled in this study. Twenty participants were subsequently excluded due to incomplete data or not meeting the inclusion criteria. As a result, the final analysis comprised 61 SCH patients and 69 HC. No significant difference was found between the SCH and HC groups in terms of age (Z = -1.740, *P* = 0.082), gender (χ^2^ = 0.448, *P* = 0.503), BMI (Z = -0.907, *P* = 0.364), or marital status (χ^2^ = 2.051, *P* = 0.891) (Table 1). Further details of the clinical data are provided in Supplemental Table 1. After quality control, the HC group yielded 7,877,295,260 clean reads and 1,128,656,046,044 clean bases, while the SCH group yielded 3,884,379,744 clean reads and 548,606,149,937 clean bases. Subsequently, the predicted gene sequences from the samples were clustered to construct a non-redundant gene set, and the abundance of genes in each sample was calculated. The HC group had 5,119,658 unique genes, and the SCH group had 3,592,898 unique genes (Supplemental Table 2).

**Table 1:** Comparison of clinical characteristics data and symptom scale assessment between the SCH group and healthy control

Parameter	HC (*n* = 69)	SCH (*n* = 61)	t/Z/χ^2^ value	*P* value
Sociodemographics				
Age [years, M (*P*_25_, *P*_75_)] ^a^	22.00 (21.00, 23.00)	25.00 (21.00, 30.00)	Z = -1.740	0.082
BMI [kg/m^2^, M (*P*_25_, *P*_75_)] ^a^	20.58 (18.82, 22.35)	20.70 (19.09, 23.95)	Z = -0.907	0.364
Gender (male/female) ^b^	35/34	32/29	χ^2^ = 0.448	0.503
Marital status (single/married) ^b^	57/12	44/17	χ^2^ = 2.051	0.152
Assessment of the severity of psychiatric symptoms				
PANSS total score [scores, mean ± SD (range)]	NA	82.77 ± 12.05 (60, 121)	-	-
PANSS positive score [scores, mean ± SD (range)]	NA	20.79 ± 4.54 (11,32)	-	-
PANSS negative score [scores, M (*P*_25_, *P*_75_)]	NA	21.00 (16.50, 24.00)	-	-

Notes: ^a^ Mann-Whitney U; ^b^ Chi-square Tests; SD, standard deviation; BMI, body mass index; Values are shown as mean ± SD. NA, not available

### The differences in the composition of archaea between SCH and HC

**Fig. 1:**
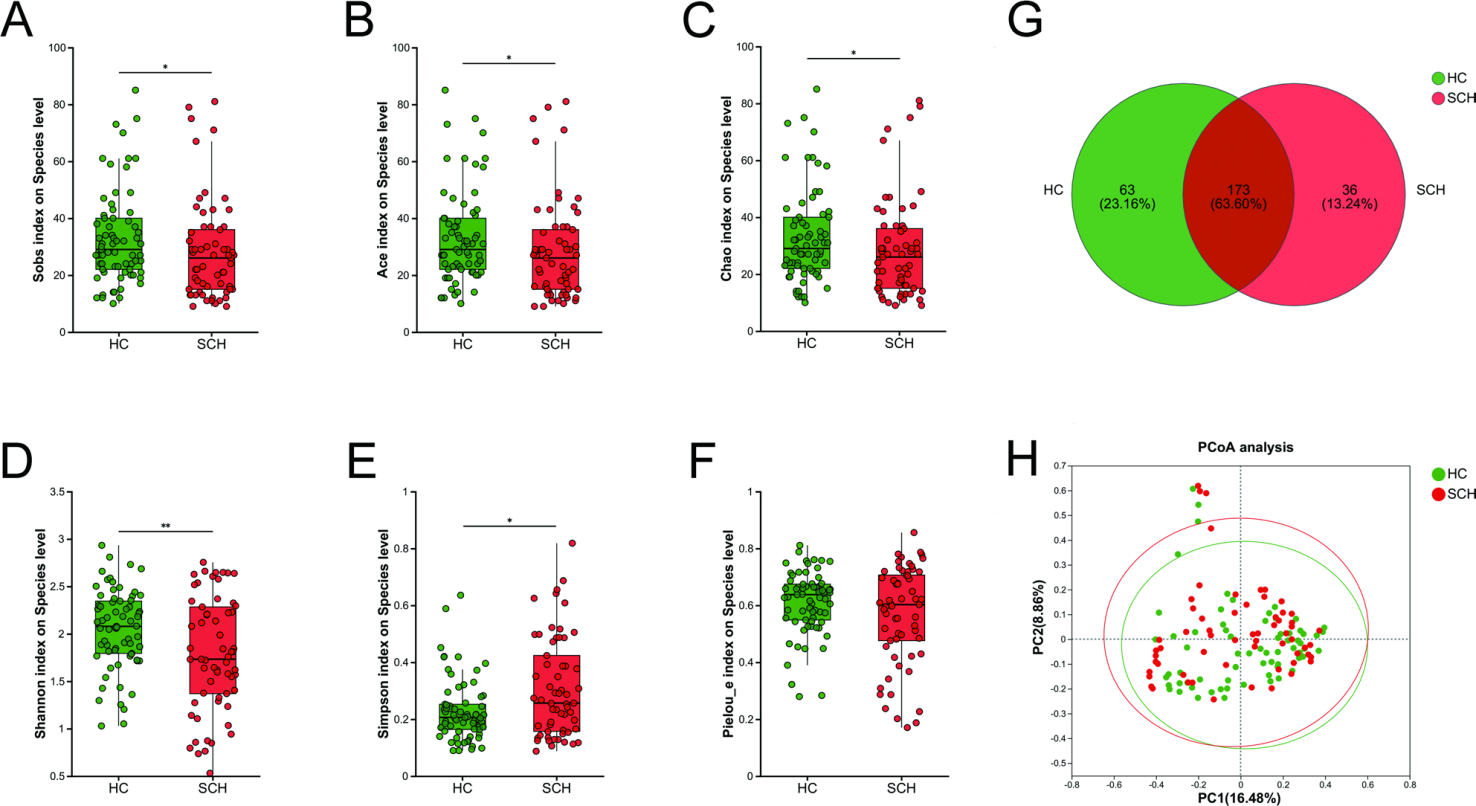
Comparison of archaeal diversity between SCH and HC groups. The boxplot reveals the difference in α-diversity represented by Sobs (**A**), Ace (**B**), Chao (**C**), Shannon (**D**), Simpson (**E**) and Pielou_e indices (**F**). (**G**) The Venn diagram shows the number of common and unique species among the SCH and HC groups. (**H**) PCoA plots of archaeal β-diversity based on Bray-curtis. The circle represents one value from an individual. PCoA, Principal coordinates analysis. * *P* < 0.05; **P < 0.01

The α-diversity metrics of archaea, including richness indices (Sobs, Ace, and Chao), community diversity indices (Shannon and Simpson), and the evenness index (Pielou_e), were compared at the species level between the SCH group and the HC group. The indices Sobs (*P* = 0.043), Ace (*P* = 0.043), Chao (*P* = 0.043), and Shannon (*P* = 0.009) were significantly lower in patients with SCH than in HC, whereas the Simpson index was higher in the SCH group (*P* = 0.019) (Fig. 1A-E). No significant differences were observed in the Pielou_e index between the two groups (Fig. 1F). A Venn diagram revealed that 173 of the 272 species were shared between the two groups, with 63 and 36 unique species in the HC and SCH groups, respectively (Fig. 1G). Principal coordinate analysis (PCoA) showed that the two groups could be distinguished at the species level, and permutational multivariate analysis of variance (PERMANOVA) also revealed significant differences between the groups based on Bray-Curtis dissimilarity (r² = 0.017, *P* = 0.012) (Fig. 1H).

**Fig. 2:**
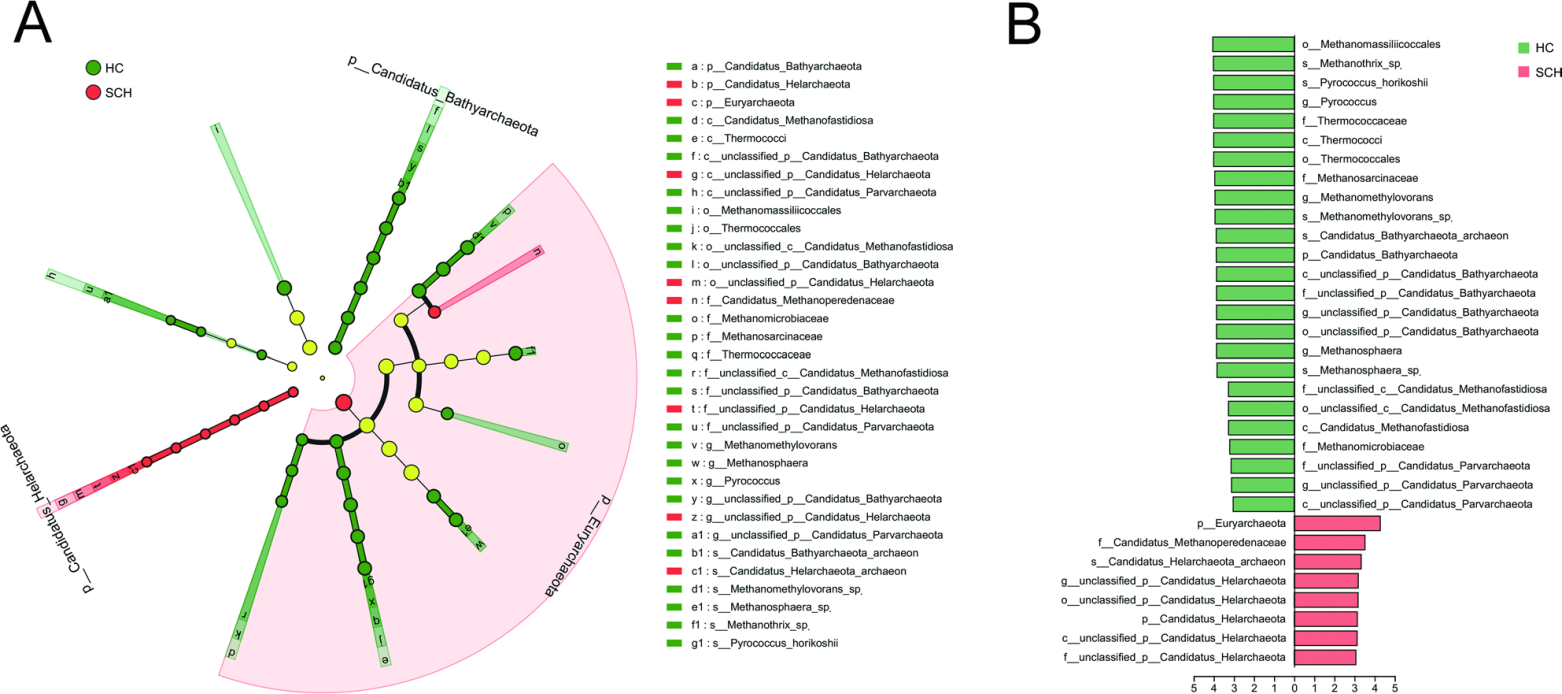
Differential taxonomic composition of gut archaea between SCH and HC groups. (**A**) The taxonomic cladogram shows the archaeal taxa enriched in HC (green dots) and SCH (red dots). Yellow dots represent microbial taxa that did not significantly affect the differences between each group. (**B**) The LDA discriminant bar chart shows the archaeal taxa with significant differences in the HC (green) and SCH (red) groups. Larger LDA scores represent a greater effect of species abundance on the different effects. Only taxa with an LDA significance threshold > 3 was presented. LDA, Linear discriminant analysis

Linear discriminant analysis (LDA) combined with effect size (LEfSe) analysis was used to validate differences in taxonomic composition between the SCH and HC groups (*P* < 0.05, LDA score > 3). At the phylum level, *Euryarchaeota* and *Candidatus_Helarchaeota* were enriched in the SCH group, while *Candidatus_Bathyarchaeota* was more abundant in the HC group. At the family level, *Candidatus_Methanoperedenaceae* and *unclassified_p_Candidatus_Helarchaeota* were more prevalent in the HC group, whereas *Thermococcaceae*, *Methanosarcinaceae*, *unclassified_p_Candidatus_Bathyarchaeota*, *unclassified_c_Candidatus_Methanofastidiosa*, *Methanomicrobiaceae*, and *unclassified_p_Candidatus_Parvarchaeota* were enriched in the SCH group. At the genus level, *Pyrococcus*, *Methanomethylovorans*, *unclassified_p_Candidatus_Bathyarchaeota*, *Methanosphaera*, and *unclassified_p_Candidatus_Parvarchaeota* were more abundant in the HC group, while *unclassified_p_Candidatus_Helarchaeota* was enriched in the SCH group. At the species level, *Methanothrix_sp.*, *Pyrococcus_horikoshii*, *Methanomethylovorans_sp.*, *Candidatus_Bathyarchaeota_archaeon*, and *Methanosphaera_sp.* were more prevalent in the HC group, whereas *Candidatus_Helarchaeota_archaeon* was enriched in the SCH group (Fig. 2A, B). Thus, the diversity and composition of gut archaea in SCH patients were significantly altered compared with those in HC.

### The differences in the composition of fungi between SCH and HC

**Fig. 3:**
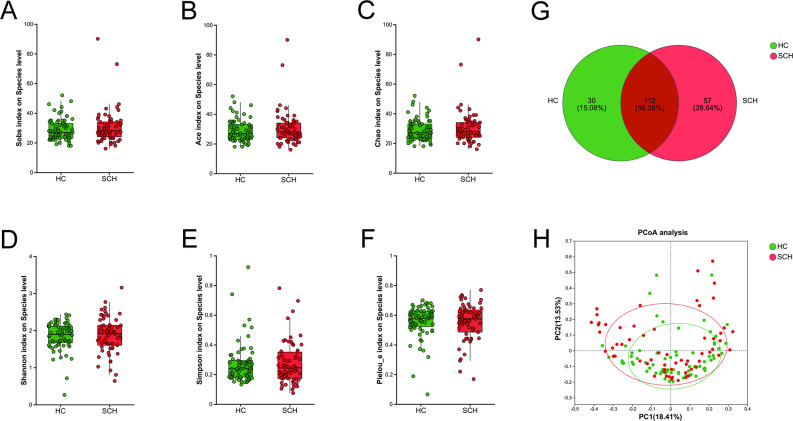
Comparison of fungal diversity between SCH and HC groups. The boxplot reveals the difference in α-diversity represented by Sobs (**A**), Ace (**B**), Chao (**C**), Shannon (**D**), Simpson (**E**) and Pielou_e indices (**F**). (**G**) The Venn diagram shows the number of common and unique species among the SCH and HC groups. (**H**) PCoA plots of fungal β-diversity based on Bray-curtis. The circle represents one value from an individual. PCoA, Principal coordinates analysis. * *P* < 0.05; **P < 0.01

**Fig. 4:**
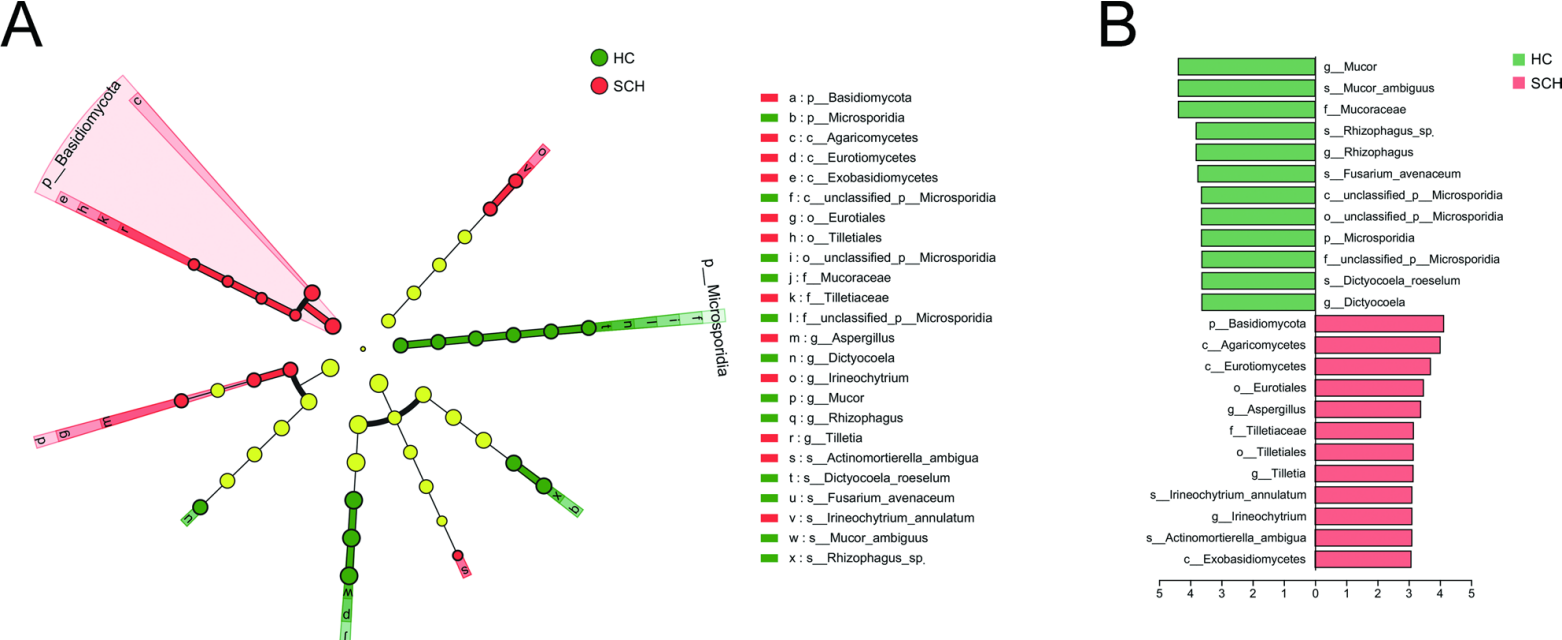
Differential taxonomic composition of gut fungi between SCH and HC groups. (**A**) The taxonomic cladogram shows the fungal taxa enriched in HC (green dots) and SCH (red dots). Yellow dots represent microbial taxa that did not significantly affect the differences between each group. (**B**) The LDA discriminant bar chart shows the fungal taxa with significant differences in the HC (green) and SCH (red) groups. Larger LDA scores represent a greater effect of species abundance on the different effects. Only taxa with an LDA significance threshold > 3 was presented. LDA, Linear discriminant analysis

No significant differences were observed in the species richness indices (Sobs, ACE, and Chao), community diversity indices (Shannon and Simpson), and evenness index (Pielou_e) of fungi between the SCH group and the HC group (all *P* > 0.05, Fig. 3A-F). A total of 112 from 199 species were shared between the two groups from a Venn diagram, providing 30 and 57 species from the HC and the SCH group, respectively (Fig. 3G). PCoA indicated that the two groups could be differentiated at the species level. PERMANOVA based on the Bray-Curtis distance also revealed significant differences between them (r^2^ = 0.017, *P* = 0.019) (Fig. 3H). Moreover, the relative abundance of gut microbiota compositions was compared by LefSe analysis (*P* < 0.05, LDA score > 3, Fig. 4A, B). At the phylum level, *Microsporidia* was enriched in the HC group, while *Basidiomycota* was enriched in the SCH group. At the family level, *Mucoraceae* and *unclassified_p_Microsporidia* were enriched in the HC group, while *Tilletiaceae* was enriched in the SCH group. At the genus level, there was a higher abundance of *Mucor*, *Rhizophagus*, and *Dictyocoela* in the HC group, while *Aspergillus*, *Tilletia*, and *Irineochytrium* were enriched in the SCH group. At the species level, there was a higher abundance of *Mucor_ambiguus*, *Rhizophagus_sp.*, *Fusarium_avenaceum*, and *Dictyocoela_roeselum* in the HC group, while *Irineochytrium_annulatum* and *Actinomortierella_ambigua* were enriched in the SCH group. These results indicated that the gut fungal composition in individuals with SCH exhibited significant differences relative to HC.

### The differences in the composition of bacteria between SCH and HC

**Fig. 5:**
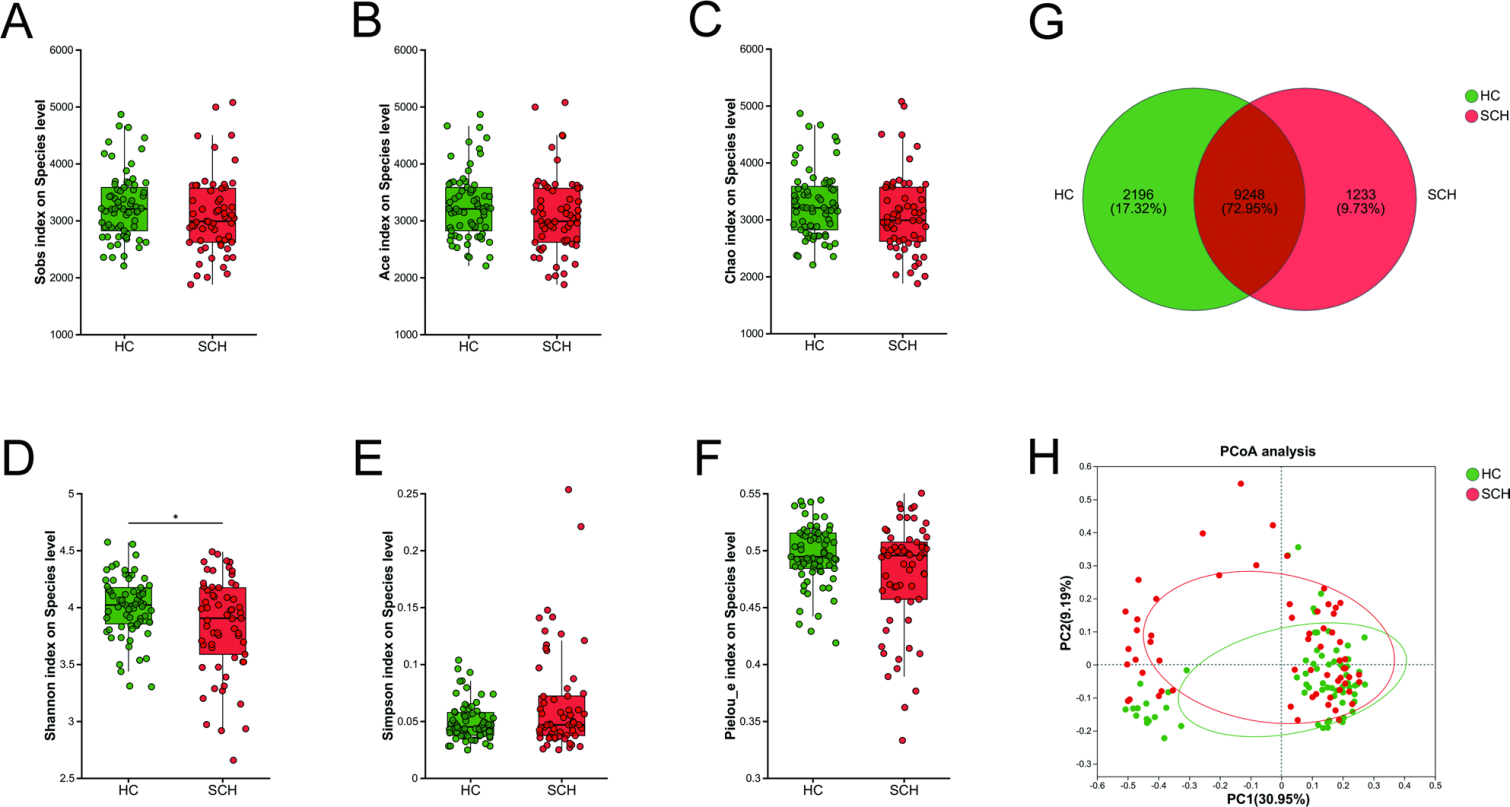
Comparison of bacterial diversity between SCH and HC groups. The boxplot reveals the difference in α-diversity represented by Sobs (**A**), Ace (**B**), Chao (**C**), Shannon (**D**), Simpson (**E**) and Pielou_e indices (**F**). (**G**) The Venn diagram shows the number of common and unique species among the SCH and HC groups. (**H**) PCoA plots of bacterial β-diversity based on Bray-curtis. The circle represents one value from an individual. PCoA, Principal coordinates analysis. * *P* < 0.05; **P < 0.01

**Fig. 6:**
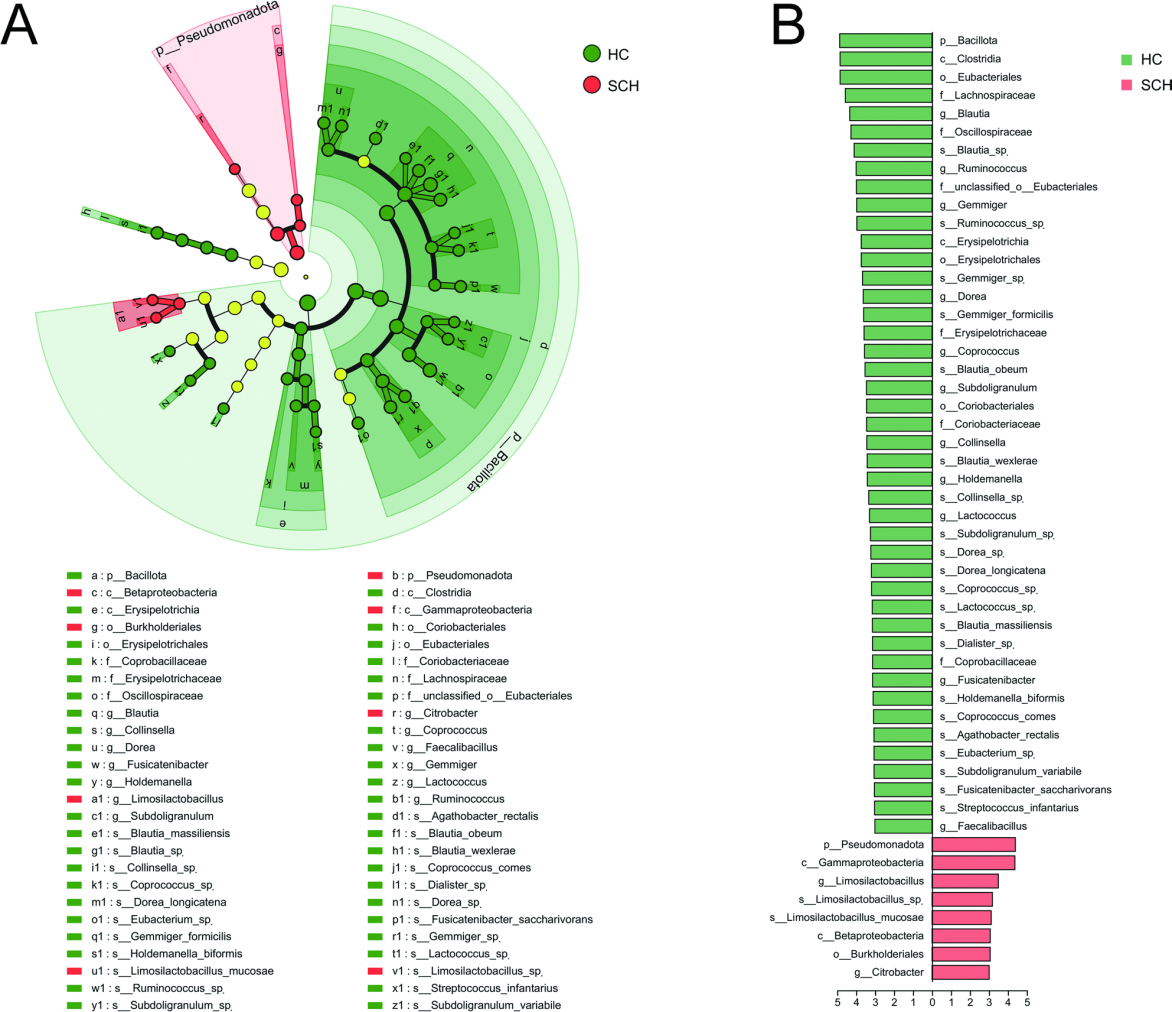
Differential taxonomic composition of gut bacteria between SCH and HC groups. (**A**) The taxonomic cladogram shows the bacterial taxa enriched in HC (green dots) and SCH (red dots). Yellow dots represent microbial taxa that did not significantly affect the differences between each group. (**B**) The LDA discriminant bar chart shows the bacterial taxa with significant differences in the HC (green) and SCH (red) groups. Larger LDA scores represent a greater effect of species abundance on the different effects. Only taxa with an LDA significance threshold > 3 was presented. LDA, Linear discriminant analysis

No significant differences were observed in the species richness indices (Sobs, ACE, and Chao) (Fig. 5A-C), community diversity indices (Simpson, Fig. 5E), and evenness index (Pielou_e, Fig. 5F) between the SCH group and the HC group (all *P* > 0.05). Whereas Shannon was lower in patients with SCH than in HC (*P* = 0.034, Fig. 5D). A total of 9248 from 12,677 species were shared between the two groups from a Venn diagram, providing 2196 and 1233 species from the HC and the SCH group, respectively (Fig. 5G). PCoA analysis demonstrated that the two groups could be distinctly differentiated at the species level (Bray-Curtis, r² = 0.024, *P* = 0.006) (Fig. 5H). Furthermore, the relative abundances of gut microbiota compositions were compared using LDA combined with LEfSe analysis. The screening criteria were established as *P* < 0.05 and an LDA score > 3. At the phylum level, *Bacillota* was enriched in the HC group, while *Pseudomonadota* was enriched in the SCH group. At the family level, *Lachnospiraceae*, *Oscillospiraceae*, *unclassified_o_Eubacteriales*, *Erysipelotrichaceae*, *Coriobacteriaceae*, and *Coprobacillaceae* were more abundant in the HC group compared to the SCH group. Additionally, 11 genera and 20 species were more prevalent in the HC group than in the SCH group. In contrast, the genera *Limosilactobacillus* and *Citrobacter*, along with the species *Limosilactobacillus_sp.* and *Limosilactobacillus mucosae*, were enriched in the SCH group (Fig. 6A, B).

### The disparity in microbial community functions between the SCH and HC

**Fig. 7:**
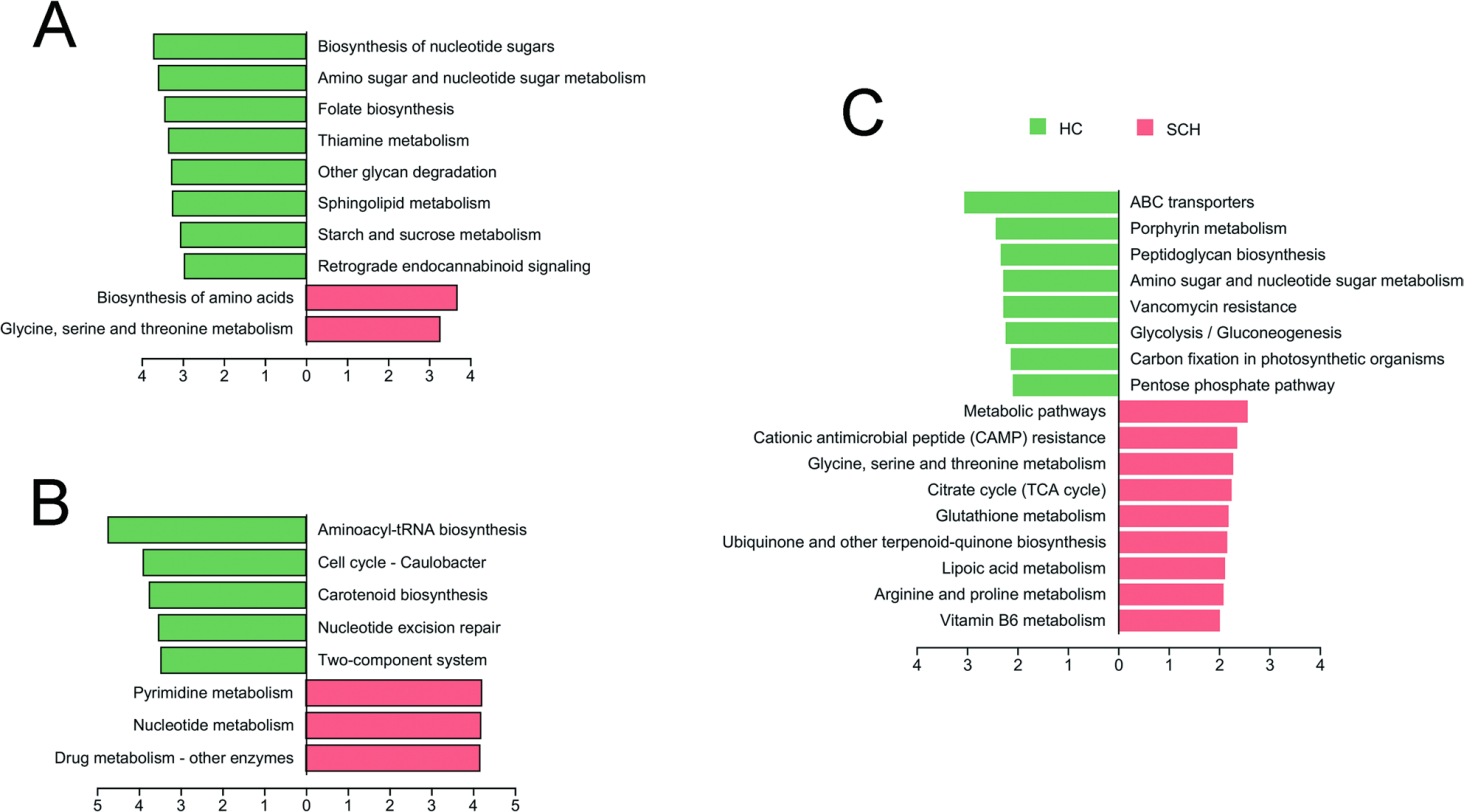
KEGG analysis showed the relative abundance of the community functions. The LDA discriminant bar chart shows the relative archaeal (**A**), fungal (**B**) and bacterial functions with significant differences enriched in the HC (green) and SCH (red) groups. Only relative functions with an LDA significance threshold > 2 was presented. KEGG, Kyoto encyclopedia of genes and genomes

The functional analysis of archaea showed that the HC group was enriched in various pathways, such as Biosynthesis of nucleotide sugars, Amino sugar and nucleotide sugar metabolism, Folate biosynthesis, Thiamine metabolism, Other glycan degradation, Sphingolipid metabolism, Starch and sucrose metabolism, and Retrograde endocannabinoid signaling. Conversely, the SCH group exhibited enrichment in the Biosynthesis of amino acids and Glycine, serine and threonine metabolism (Fig. 7A). The functional analysis of fungi further indicated that the HC group was enriched in Aminoacyl-tRNA biosynthesis, Cell cycle - Caulobacter, Carotenoid biosynthesis, Nucleotide excision repair, and Two-component system. In contrast, the SCH group was enriched in Pyrimidine metabolism, Nucleotide metabolism, and Drug metabolism - other enzymes (Fig. 7B). The functional analysis of bacteria revealed that the HC group was enriched in ABC transporters, Peptidoglycan biosynthesis, Porphyrin metabolism, Amino sugar and nucleotide sugar metabolism, Vancomycin resistance, Glycolysis / Gluconeogenesis, Carbon fixation in photosynthetic organisms, and Pentose phosphate pathway. In contrast, the SCH group was enriched in Metabolic pathways, Cationic antimicrobial peptide (CAMP) resistance, Glycine, serine and threonine metabolism, Citrate cycle (TCA cycle), Glutathione metabolism, Ubiquinone and other terpenoid-quinone biosynthesis, Lipoic acid metabolism, Arginine and proline metabolism, and Vitamin B6 metabolism (Fig. 7C).

### Gut microbial biomarkers distinguishing SCH from HC and their correlation with clinical parameters

**Fig. 8:**
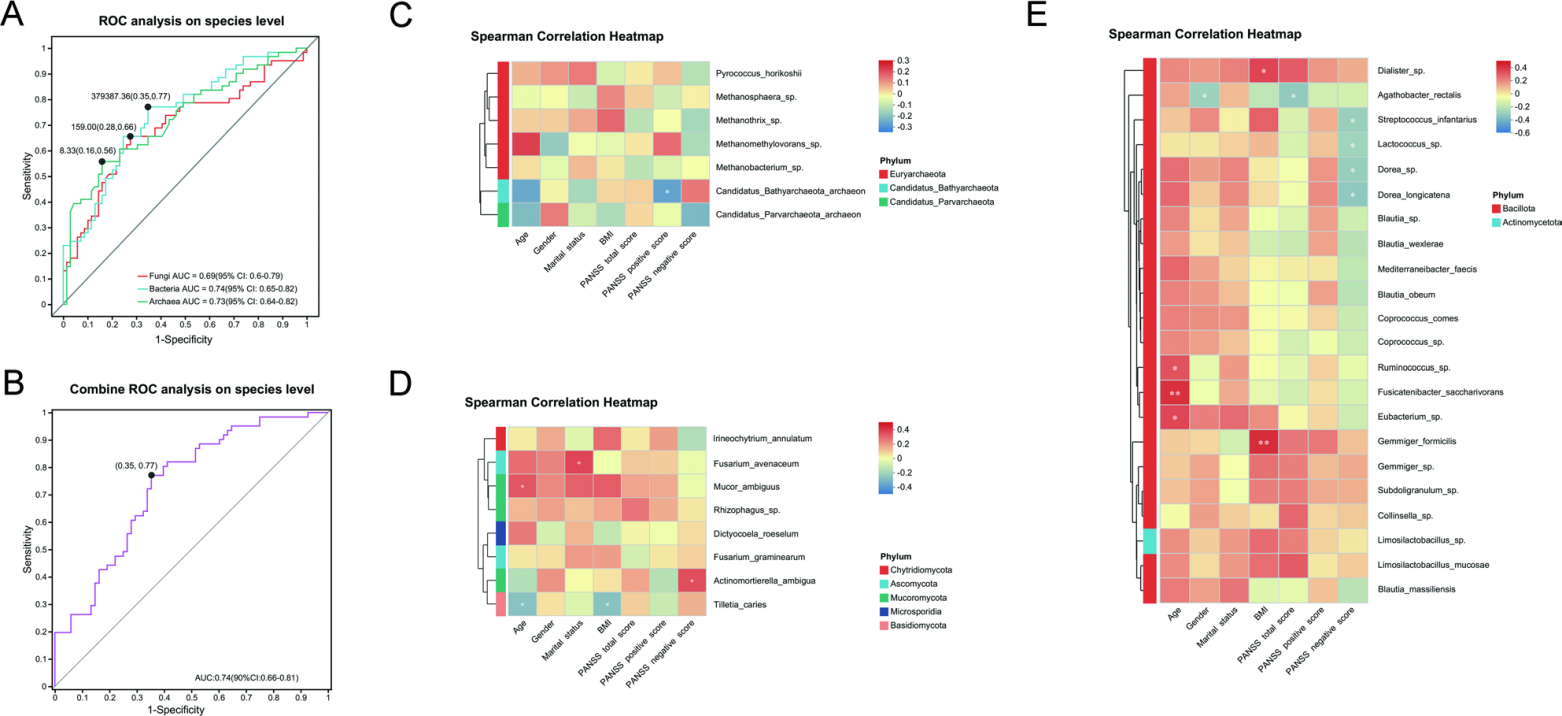
The microbial biomarkers distinguishing SCH from HC and their correlation with clinical parameters. (**A**) AUC value of ROC analysis reflects the differential diagnostic potential of archaea (green curve), fungi (red curve) and bacteria (blue curve) for discriminating SCH and HC. (**B**) AUC value of ROC analysis reflects the diagnostic potential of combined panel of all 39 microbes (pink curve) for discriminating SCH and HC. Y-axis represents the value of sensitivity; the X-axis shows the value of 1 − specificity. The heatmap illustrates Spearman’s rank correlation coefficients between clinical parameters (including age, gender, marital status, BMI, PANSS total score, PANSS positive score, and PANSS negative score) and differentially abundant archaeal (**C**), fungal (**D**), and bacterial species that were enriched in SCH. * *P* < 0.05; **P < 0.01; ***P < 0.001

LEfSe analysis (LDA ≥ 3) identified 9 archaea, 8 fungi, and 22 bacteria as differential taxa at the species level. The diagnostic potential of these microbes was assessed using the area under the receiver operating characteristic curve (AUC). The AUC values for the archaea, fungi, and bacteria were 0.73, 0.69, and 0.74, respectively, indicating their ability to distinguish SCH from HC (Fig. 8A). A combined panel of all 39 microbes achieved an AUC of 0.74, similar to that of the bacterial panel alone (Fig. 8B). Additionally, the relative abundance of *Candidatus Bathyarchaeota archaeon* was negatively correlated with the PANSS positive score (Fig. 8C). The abundance of fungi *Actinomortierella_ambigua* was positively correlated with the PANSS negative score (Fig. 8D). Moreover, the relative abundance of *Streptococcus infantarius*, *Lactococcus_sp*., *Dorea_sp.*, and *Dorea_longicatena* was negatively correlated with the PANSS negative score (Fig. 8E).

## Discussion

Gut microbial dysbiosis in schizophrenia (SCH) is increasingly recognized as a driver of neuroinflammatory and neurochemical disruptions, underscoring the role of the gut-brain axis in the disease [40, 41]. While bacteria are abundant members of the gut microbiome, archaea and fungi, which are in the minority, also play important roles in intestinal microecological balance and body metabolism. In this study, we comprehensively compare the compositional and functional differences of gut archaea, fungi, and bacteria between individuals with SCH and healthy controls (HC) by using metagenomic techniques. Additionally, we established potential gut microbial biomarkers at the species level that may suggest as indicators for distinguishing SCH from HC. These findings revealed that the dysbiosis of the microbiota, including the minor microbial groups such as archaea and fungi, may also be involved in the pathogenesis of schizophrenia.

Archaea account for 1% to 2% of human gut microbes and are mostly strict anaerobes [42]. It represents a distinct domain of life, characterized by unique cell walls and membranes and specialized enzymes and metabolic pathways [43]. Despite being in its infancy, research on gut archaea has yielded compelling evidence that these microorganisms significantly contribute to gut microecological balance and health maintenance, host metabolism regulation, and immune function modulation [44, 45]. For example, methanogens are the primary archaea in the human gastrointestinal tract. These archaea may mitigate the production of ROS and TMAO, as well as intestinal permeability [46, 47]. Additionally, methane produced by methanogens can indirectly regulate the antioxidant response [48, 49]. Given the extensive reports of excessive inflammatory responses and oxidative stress in SCH [50–52], we speculate that alterations in the composition and function of gut archaea may also be present in this disorder. However, to the best of our knowledge, no studies have yet reported changes in gut archaea in patients with SCH. The α-diversity of gut archaea is influenced by geographical environment and lifestyle and has important implications in health and disease. For example, the alpha diversity of archaea is higher in healthy people, while it is significantly reduced in certain disease states, such as inflammatory bowel disease (IBD) and obesity [53]. Consistent with this finding, we observed no significant difference in the evenness of archaeal distribution between the SCH and HC groups. However, the richness and community diversity of archaea were significantly reduced in the SCH group. Similarly, significant differences in β-diversity were also observed between the two groups, which strongly suggests that the archaeal diversity in SCH is significantly altered. We further compared the differences in archaeal composition between the two groups at each taxonomic level and found significant differences at the phylum, family, order, genus, and species levels. Of note, species such as *Pyrococcus horikoshii* and *Candidatus* Bathyarchaeota archaeon were enriched in the HC group. A negative correlation was observed between the abundance of *Candidatus* Bathyarchaeota archaeon and the PANSS positive score, suggesting a potential link to clinical symptoms. While a recent study identified its capacity to synthesize polyethylene terephthalate-active enzymes [54], the physiological role of this archaeon in gut ecology and its possible connection to SCH pathophysiology are still unclear and merit further exploration.

In addition, functional analysis showed that the enrichment of glucose and lipid metabolic pathways was decreased, while the enrichment of amino acid synthesis and some amino acid metabolic pathways (glycine, serine and threonine) was increased in the SCH group. Glucose metabolism and lipid metabolism serve as fundamental pathways for maintaining bodily functions, whereas amino acid metabolism is associated with neurotransmitter synthesis and oxidative stress. Previous research has indicated that glucose metabolism abnormalities emerge in the early stages of SCH, and the interplay between these metabolic issues and white matter connectivity abnormalities may significantly contribute to cognitive impairments in SCH [55]. Meanwhile, the role of amino acids, such as glycine and serine, in the treatment of SCH has also been reported [56, 57]. Furthermore, the enrichment of retrograde endocannabinoid signaling and folate biosynthesis pathways were also reduced in the SCH group. The endocannabinoid system is a key mediator of brain-gut axis communication, and its involvement in SCH has been documented [58, 59]. Moreover, research showed that individuals with SCH have lower serum folate levels and suggests that folate supplementation may be beneficial in the treatment of SCH [60]. Together, these findings point to a discernible alteration in the diversity and composition of gut archaea in SCH, alongside functional changes in metabolic pathways. While preliminary, these functional shifts (e.g., in folate and energy metabolism) could represent a novel layer of interaction with host physiology, potentially relating to established aspects of SCH pathophysiology such as oxidative stress or neuroinflammation.

The identification of a distinct archaeal signature in schizophrenia (SCH) carries considerable scientific weight. It fundamentally expands the scope of gut dysbiosis in SCH beyond bacteria, pointing to a more pervasive disruption of the gut ecosystem. The observed alterations, particularly among methanogens, may exert functional consequences relevant to SCH pathophysiology, potentially via influences on key metabolic pathways such as butyrate and histidine metabolism [61]. which are implicated in immune and neuro-immune regulation. Furthermore, the archaeal biomarker panel demonstrated discriminatory power comparable to its bacterial counterpart, highlighting its promise as a novel and stable diagnostic tool. While these findings are primarily hypothesis-generating due to the inherent challenges in culturing gut archaea and delineating their mechanisms, they chart a clear course for future research. Critical next steps will include correlating archaeal abundance with host inflammatory markers and employing fecal microbiota transplantation (FMT) in animal models to transition from correlation to causation, ultimately unraveling the role of this enigmatic domain in the gut-brain axis of SCH.

Fungi are detectable throughout the intestinal and colon segments in adults, comprising approximately 0.1% of gut microbial DNA [62]. While fungi can cause infections, they also contribute to gut and systemic homeostasis [63]. Notably, fungi can transition from commensal to pathogenic states through morphological shifts, interactions with gut bacteria, host immune responses, and genetic/epigenetic factors [64, 65]. Despite the fungal component, or mycobiome, in SCH being less explored, emerging evidence suggests a potential link between gut mycobiome dysbiosis and SCH development. Zhang et al. were the first to investigate changes in gut fungal alterations, revealing that the gut mycobiota in SCH patients exhibit reduced α-diversity and compositional changes, such as elevated *Chaetomium* and diminished *Trichoderma* levels relative to HC [66]. Yuan et al. used 18 S rRNA gene amplicon sequencing to profile gut mycobiota and found reduced fungal α-diversity and a significantly lower fungi-to-bacteria α-diversity in drug-naïve, first-episode SCH patients compared with that of HC [67]. Subsequently, they used internal transcribed spacer (ITS) sequencing to profile gut mycobiota in 109 chronic SCH and 77 HC and observed a reduced fungal diversity and a depletion of fungi, such as *Saccharomyces cerevisiae* in SCH, suggesting fungal dysbiosis may contribute to inflammation [68]. The present study revealed alterations in fungal composition in SCH patients, and the differences in fungal function between SCH and HC were mainly reflected in microflora balance, nucleotide metabolism, and aminoacyl-tRNA biosynthesis. These findings suggest that dysfunction of the mycobiome is one of the manifestations of microbial dysbiosis in SCH, and the abnormal biological functions mediated by these changes may also be involved in the pathogenesis of the disorder. Inconsistent with these results, we found no significant difference in fungal α-diversity between SCH and HC, although a difference in β-diversity was observed. Meanwhile, the fungal differences we identified were also inconsistent with prior reports. These conflicting results may be related to differences in patient characteristics (first or recurrent, acute or chronic), fecal preservation, and fungal detection methods [69]. Moreover, there was a higher abundance of species *Fusarium_avenaceum* and *Dictyocoela_roeselum* in the HC group. However, *Irineochytrium_annulatum* and *Actinomortierella ambigua* were enriched in the SCH group, and the abundance of *Actinomortierella ambigua* was positively correlated with the PANSS negative score. However, the role of the above fungi in the gut has not been reported, and their function warrants further investigation.

Compared with archaea and fungi, the number of studies examining differences in bacterial composition between SCH patients and normal controls is significantly larger. Most α-diversity analyses revealed no statistically significant changes, whereas β-diversity analysis indicated significant differences in patients with SCH. Similar to this result, we found no significant difference in the α-diversity index between SCH and HC groups except for the Shannon index. Moreover, PCoA analysis unveiled pronounced differences in β-diversity at the species level between these two groups. While our results do not robustly endorse the notion that the diversity and richness of gut bacteria in SCH patients markedly diverge from those in HC individuals, the gut microbial composition of SCH patients was found to be significantly distinct from that of the healthy population at the species level. Considering that the study of the bacterial structure, especially at the species level, and its function is still insufficient [70], the present study compared the differences between the two groups of microflora at the species level and analyzed the functional differences accordingly. We found that 20 species were more prevalent in the HC group, and the abundance of *Streptococcus infantarius*, *Lactococcus_sp.*, *Dorea_sp.* and *Dorea_longicatena* was negatively correlated with PANSS negative score. *Streptococcus infantarius*, *Dorea_sp.* and *Dorea longicatena* play an important role in the intestinal microecosystem and help to maintain intestinal microecological balance [71–73]. Whereas *Lactococcus_sp.* a lactic acid bacterium, is involved in nutrient metabolism and immune regulation [74, 75]. In contrast, species *Limosilactobacillus_sp.* and *Limosilactobacillus_mucosae* were enriched in the SCH group. Previous studies showed that *Limosilactobacillus_sp.* may contribute to acetic acid production [76], whereas *Limosilactobacillus mucosae* has been shown to alleviate dextran sodium sulfate-induced colitis by the inhibition of NF-κB and STAT3 signaling [77]. Thus, alterations in bacterial abundance may influence gut microecology, potentially contributing to the pathogenesis of SCH. However, the impact of individual bacterial species on mental symptoms requires further investigation. In addition, functional analysis showed that the amino acid, glutathione and lipoic acid metabolic pathway was enriched and the glucose metabolism was decreased in the SCH group. It is suggested that the abnormal function of bacterial flora, which leads to abnormal peripheral metabolism [78–80], may be one of the potential causes of SCH. Interestingly, functional analysis revealed a consistent enrichment of glycine, serine, and threonine metabolism in the SCH group, a finding also observed in the archaeal functional profile. This convergence suggests a potential link between this metabolic pathway and schizophrenia, underscoring a possible role shared by both archaea and bacteria in the disorder. It is noteworthy that glutathione, a critical antioxidant, is a tripeptide composed of glutamate, cysteine, and glycine. Deficits in the glutathione redox cycle have been documented in patients with SCH [81], and glycine and serine themselves have been implicated in the pathogenesis of the illness [57, 82]. Taken together, these observations support the hypothesis that gut microbiota dysbiosis may contribute to schizophrenia symptomatology through disruptions in amino acid metabolism. However, the precise mechanistic details underlying this relationship remain to be elucidated.

Additionally, we identified a panel of 9 archaea, a panel of 8 fungi and a panel of 22 bacteria capable of distinguishing SCH patients from HC. The efficacy of bacteria at the species level (AUC = 0.74) is similar to that obtained in previous studies at genus level (AUC = 0.739) [67]. Moreover, the bacterial panel exhibited the highest efficacy, while the fungal panel demonstrated the lowest efficacy. These findings suggest that microbial markers identified at the species level may play a significant role in distinguishing SCH, with bacterial markers being particularly important for this purpose. Notably, the archaeal panel (AUC = 0.73) exhibited a comparable efficacy to bacteria, suggesting that archaea may play an important role in the identification of schizophrenia. Nevertheless, results of previous studies on gut bacteria in SCH exhibited high heterogeneity, mainly attributable to variations in patient populations, including sample size, disease severity and course, gender and age, dietary habits, living area and cultural differences, medication use, and diagnostic criteria [83, 84]. The validity of the above panels needs to be verified by large sample cohort studies.

Several limitations of the present study should be noted. First, the sample size appears relatively small, and there were no discovery or validation sets to further verify the microbial markers. Second, although we identified some unreported differential microbial species (such as archaea and fungi), and the relative abundance of some microbes was correlated with certain symptoms in patients, the roles of these microbes in the gut remain unclear. Their contribution to the explanation of the pathogenesis of schizophrenia is limited. Additionally, this study is a cross-sectional study and did not take into account the impact of pharmacological interventions on the microbiota. Further validation of the findings with larger sample sizes in fecal metagenomic studies is still needed.

## Conclusions

In summary, this study describes distinct imbalances in the gut archaeal, fungal, and bacterial communities and their associated functions in individuals with schizophrenia (SCH). We also identified preliminary panels of archaeal (9 species, AUC = 0.73), fungal (8 species, AUC = 0.69), and bacterial (22 species, AUC = 0.74) species that, in this cohort, showed modest ability to differentiate SCH patients from healthy controls (HC). These results offer exploratory evidence for the potential of microbial biomarkers and contribute to the growing foundation for understanding the microecological mechanisms in SCH.

## Supplementary Information

Below is the link to the electronic supplementary material.

Supplementary Material 1

Supplementary Material 2

## Data Availability

The data supporting this study’s findings are available from the corresponding author upon reasonable request.
